# A nomogram for predicting lymph nodes metastasis at the inferior mesenteric artery in rectal cancer: a retrospective case–control study

**DOI:** 10.1007/s13304-023-01748-5

**Published:** 2024-01-21

**Authors:** Chunhao Xu, Qiaoyi Huang, Yunhuang Hu, Kai Ye, Jianhua Xu

**Affiliations:** 1 Department of Gastrointestinal Surgery, The Second Affiliated Hospital of Fujian Medical University, Quanzhou, Fujian 362000 China; 2 Department of Gynaecology and Obstetrics, The Second Affiliated Hospital of Fujian Medical University, Quanzhou, Fujian 362000 China

**Keywords:** Colorectal cancer·No. 253 lymph nodes, Lymph nodes at the inferior mesenteric artery, Risk factor, Nomogram

## Abstract

According to past and current literature, metastasis of the lymph nodes at the inferior mesenteric artery (IMA-LN), also known as 253LN of colorectal cancer has been seldom investigated. To date, there are still controversies on whether the 253LN need to be routinely cleaned. Using specific criteria, 347 patients who underwent radical resection for rectal cancer between April 2019 and July 2022 were selected for the study. Logistic regression was used to determine the likelihood that a patient may suffer 253LN metastasis, and a nomogram for 253LN metastasis subsequently developed. The c-index and calibration curve were used to evaluate precision and discrimination in the nomogram, and the appropriateness of the final nomogram for the clinical setting determined using decision curve analysis (DCA). 253LN metastases appeared in the pathological specimens of 29 (8.4%) of the selected patients. Logistic regression showed that preoperative parameters including serum carcinoembryonic antigen (CEA) value ( > 5 ng / ml, OR = 2.894, *P* = 0.023), distance from anal margin (> 9 cm, OR = 2.406, *P* = 0.045) and degree of differentiation (poor, OR = 9.712, *P* < 0.001) were significantly associated with 253LN metastasis. A nomogram to predict 253LN metastasis in rectal cancer was developed and showed considerable discrimination and good precision (c-index = 0.750). Furthermore, DCA confirmed that the nomogram has some feasibility for the clinical environment. Clinicopathological and radiological patient data can be pivotal for making surgical decisions relating to 253LN metastasis. A nomogram was developed using this data, providing an objective method that can significantly improve prognoses in colorectal cancer.

## Introduction

Colorectal cancer is the third most common cancer in the world and the second leading cause of cancer-related death [1]. Endorsed by gastroenterologists worldwide, total mesorectal excision (TME) is a widely accepted surgical method for rectal cancer, and is proven to significantly improve prognoses [2]. This method involves the dissection of lymph nodes (LN) and blood vessels in the mesorectal envelope, thus considerably reducing the likelihood of recurrence. The LN at inferior mesenteric artery (IMA-LN, also known as 253LN), were generally classified as D3 regional LN in Japan, which was usually described as LN located along the IMA near the origin of the left colonic artery [3]. Some studies have reported that 253LN positivity is a robust predictor of postoperative recurrence and prognosis of colorectal cancer [4]. According to literature the metastasis rate of 253LN is low, ranging between 0.3% and 14.2% [5]. However, 253LN dissection may reduce perfusion around the anastomotic site and increase the possibility of anastomotic leakage. Damage to the autonomic nerve located near the origin of the IMA is also likely, an injury that can manifest in impaired urination or sexual dysfunction [6]. Some studies even argued that 253 LN dissection has no survival benefit for patients [7]. Therefore, the value of 253 LN dissection remains controversial, and the clinical evidence for the long-term benefits of 253LN dissection remains insufficient [8].

The aim of this study was to develop a nomogram for accurate and effective prediction of 253LN metastases in rectal cancers. A retrospective analysis of risk factors for metastasis steered the development process, ensuring that the surgical decisions made using the nomogram have strong statistical rationale and are reliable. Unnecessary over dissection of LN and possible complications are consequently avoided, giving the afflicted patients better prognoses.

## Methods

### Patient selection

**Fig. 1:**
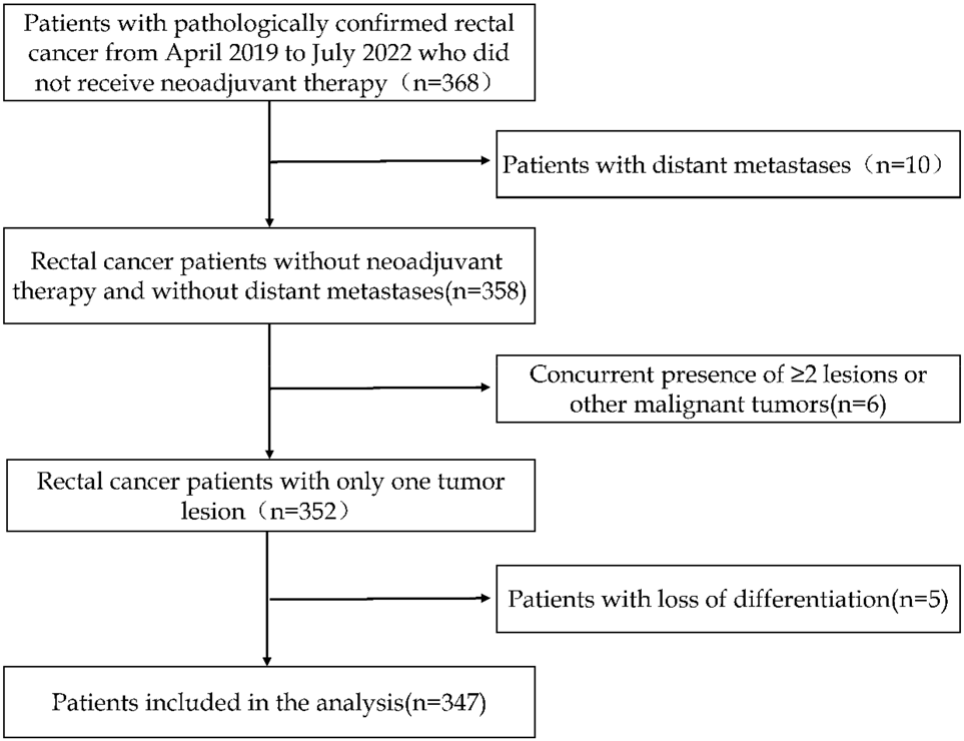
Cases selection flow chart

This is a retrospective study based on clinical databases of our center. The study was approved by the ethics committee of the Second Affiliated Hospital of Fujian Medical University (2022-341). 253LN positive (253LN +) indicates the presence of at least one metastatic LN in the pathological specimens from the 253LN region. A total of 347 rectal cancer patients who underwent radical surgery in the department of gastrointestinal surgery from April 2019 to July 2022 were selected for this study. The inclusion criteria were as follows: (1) the primary tumor was located in the rectum; (2) Underwent radical resection of rectal cancer; (3) Postoperative histopathology confirmed that rectal cancer (4) 253LN was isolated and sent for examination. The exclusion criteria were as follows: (1) distant metastasis, (2) received neoadjuvant therapy, (3) emergency or palliative surgery, (4) 2 or more primary tumor lesions, (5) Incomplete data. A decision flowchart was created to aid the patient selection process (Fig. 1).

### Surgical intervention

**Fig. 2:**
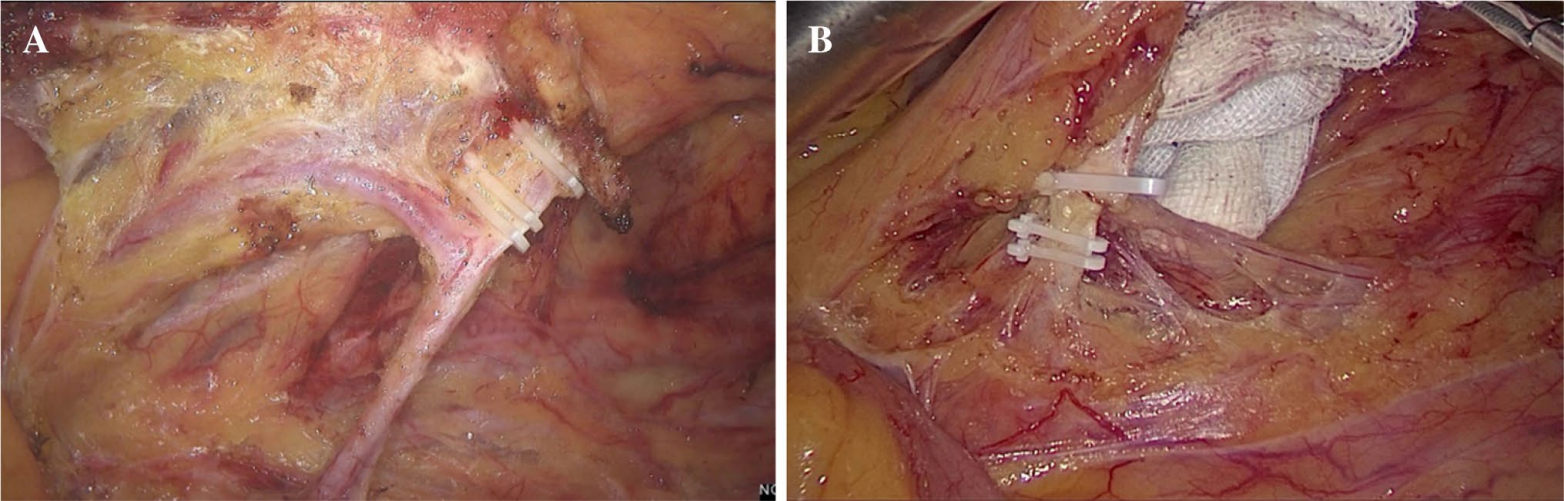
Intraoperative imaging. A Low ligation: ligate the artery at the distal end of the left colonic artery branch. B High ligation: ligate the artery about 1 cm away from the root of the inferior mesenteric artery.

All the selected patients were subjected to laparoscopic surgery by the same surgical team, which boasted extensive laparoscopic experience. All radical resection of rectal cancer was performed according to the principles of TME. In laparoscopic radical resection of rectal cancer, the ligation methods of IMA were divided into two types, one was high ligation (HL) and the other is low ligation (LL), the two methods were shown in Fig. 2. All patients underwent 253LN dissection, and 253LN biopsy performed to ensure that each pathological report has a description of 253LN status.

### Patient profiles

A total of 17 possible preoperative predictors were identified from the patient profiles and listed. We convert continuous data into binary data. These included age (≤ 75 years and > 75 years), sex (female and male), hypertension, smoking history, diabetes, preoperative obstruction, body mass index (BMI) (≤ 24 kg/m^2^ and > 24 kg/m^2^), neutrophil-to-lymphocyte ratio (NLR) (≤ 3.4 and > 3.4), preoperative albumin (≤ 47 g/L and > 47 g/L), preoperative carcinoembryonic antigen (CEA) (≤ 5 ng/ml and > 5 ng/ml), preoperative carbohydrate antigen 199 (CA19-9) (≤ 33 µ/ml and > 33 µ/ml), the distance from the lower edge of the lesion to the anus (≤ 9 cm and > 9 cm), the lesion occupies the circumference of the bowel (≤ 1/2 and > 1/2), tumor size (≤ 3.5 cm and > 3.5 cm), pathological type (adenocarcinoma and signet ring cell carcinoma), differentiation (poor and well/moderate), T stage (T1–2 and T3–4). In addition, three nonpredictive indicators were considered: operative time, intraoperative blood loss, number of LN dissections.

### Definition of 253 LN

The IMA-LN is defined as a LN that runs along the IMA from the origin of the IMA to the bifurcation with the left colonic artery [9].

### Statistical inference

Statistical analysis was performed using IBM SPSS software (version 27.0). *T* test was adopted for the measurement data conforming to normal distribution, and the Mann–Whitney *U* test was adopted for the measurement data not conforming to normal distribution. Chi square test was performed to compare the count data. The best cut-off values of BMI, NLR, preoperative albumin, CEA, CA19-9, distance from anal verge, and tumor size for predicting 253LN metastasis were computed using the Youden’s index of a receiver operating characteristic (ROC) curve [10]. Variables with *P* < 0.1 obtained from the comparison between groups were included in the logistic regression to determine the independent risk factors for 253LN metastasis. A nomogram for predicting 253LN metastasis was constructed using R version 4.2.1. and its statistical robustness ascertained.

The nomograph was verified internally using bootstrap resampling, and the performance evaluated using the c-index and calibration curve, the former indicating the degree of discrimination in the nomogram. A calibration curve was used to represent the relationship between the observation frequency and the prediction probability. The clinical value of the developed nomogram was evaluated using decision curve analysis (DCA) by quantifying the net benefit for each possible threshold probability.

## Results

### Clinical profiles and outcomes

Of the 347 patients selected for the study, 29 (8.4%) had 253LN metastasis. There was no difference in the number of retrieved LN in the 253 LN ( +) group and the 253 LN ( −) group (22.3 ± 10.4 vs. 19.7 ± 8.2, *P* = 0.284). In the T stage of the metastasis group, no case was at the T1 stage (0%), 2 cases were at the T2 stage (6.9%), 22 cases at T3 stage (75.9%), 5 cases at T4 stage (17.2%). As evidenced in the biopsy results, majority of the patients exhibited stage T3 and T4 growth, indicating that the metastases had spread to the outer lining of the bowel. In the metastatic group, 8 cases were poorly differentiated (27.6%), 21 cases were moderately differentiated (72.4%) and no case was highly differentiated (0%). Intergroup analysis showed that preoperative serum CEA, preoperative serum CA19-9 and tumor differentiation were closely related to 253LN metastasis (*P* < 0.05). Other clinical characteristics were similar between the two groups. Patients with CEA > 5 ng/ml had a higher incidence of 253LN metastasis (72.4% vs. 45.3%, *P* = 0.005). The incidence of 253LN metastasis was higher in patients with poor pathological differentiation than in patients with moderate pathological differentiation (27.6% vs. 4.1%, *P* < 0.001). The incidence of 253LN metastasis was higher in patients with a distance from anal verge > 9 cm (65.5% vs. 47.8%, *P* = 0.068). The clinical characteristics of the patients are summarized in Table 1. Postoperative complications occurred in 27 patients (7.8%). The complications suffered by most of these patients ranged between grades II-IV of the Clavien–Dindo classification of surgical complications. Eight of the twenty-seven patients developed two or more complications. The incidence of postoperative complications were early postoperative small bowel obstruction (5.8%, *n* = 20), anastomotic leakage (2.3%, *n* = 8), anastomotic bleeding (2.0%, *n* = 7), and incision infection (1.4%, *n* = 5).

**Table 1:** Characteristics of patients

Characteristics	Total(*n* = 347)	Non-metastasis *n* = (318)	Metastasis (*n* = 29)	*P*
Age, (*n*%)				1.000
≤ 75 years	300 (86.5)	275 (86.5)	25 (86.2)	
> 75 years	47 (13.5)	43 (13.5)	4 (13.8)	
Sex, (*n*%)				0.272
Male	201 (57.9)	187 (58.8)	14 (48.3)	
Female	146 (42.1)	131 (41.2)	15 (51.7)	
Hypertension, (*n*%)	81 (23.3)	72 (74.2)	9 (31.0)	0.306
Smoking history, (*n*%)	38 (11.0)	36 (11.3)	2 (6.9)	0.755
Diabetes, (*n*%)	41 (11.8)	38 (10.9)	3 (10.3)	1.000
Preoperative obstruction, (*n*%)	37 (10.7)	33 (10.4)	4 (13.8)	0.532
BMI, (*n*%)				0.258
≤ 24 kg/m^2^	247(71.2)	229 (72.0)	18 (62.1)	
> 24 kg/m^2^	100 (28.8)	89 (28.0)	11 (37.9)	
NLR, (*n*%)				0.202
≤ 3.4	282(81.3)	261 (82.1)	21 (72.4)	
> 3.4	65 (18.7)	57 (17.9)	8 (27.6)	
Preoperative albumin, (*n*%)				0.109
≤ 47 g/L	291 (83.9)	270 (84.9)	21 (72.4)	
> 47 g/L	56 (16.1)	48 (15.1)	8 (27.6)	
CEA, (*n*%)				0.005
≤ 5 ng/ml	182(52.4)	174 (54.7)	8 (27.6)	
> 5 ng/ml	165(47.6)	144 (45.3)	21 (72.4)	
CA19-9, (*n*%)				0.03
≤ 33 µ/ml	292 (84.1)	272 (85.5)	20 (69.0)	
> 33 µ /ml	55 (15.9)	46 (14.5)	9 (31.0)	
Operative time (min)	164.9 ± 47.8	166.3 ± 48.1	161.2 ± 44.1	0.590
Blood loss (ml)	53.4 ± 148.9	54.7 ± 155.1	39.3 ± 38.8	0.539
Number of retrieved LNs	19.9 ± 8.4	19.7 ± 8.2	22.3 ± 10.4	0.284
Distance from anal verge, (*n*%)				0.068
≤ 9 cm	176(50.7)	166(52.2)	10 (34.5)	
> 9 cm	171 (49.3)	152 (47.8)	19 (65.5)	
Occupied rectal circumference, (*n*%)				0.391
≤ 1/2	182(52.4)	169 (53.1)	13 (44.8)	
> 1/2	165 (47.6)	149 (46.9)	16(55.2)	
Tumor size, (*n*%)				0.475
≤ 3.5 cm	129 (37.2)	120 (37.7)	9 (31.0)	
> 3.5 cm	218 (62.8)	198 (62.3)	20 (69.0)	
Pathological type, (*n*%)				0.220
Adenocarcinoma	337 (99.1)	311 (99.4)	26 (96.3)	
Signet ring cell carcinoma	3 (0.9)	2 (0.6)	1 (3.7)	
Differentiation, (*n*%)				< 0.001
Poor	21 (6.1)	13 (4.1)	8 (27.6)	
Well/moderate	326 (93.9)	305 (95.9)	21(72.4)	
T stage, (*n*%)				0.088
T1–2	65 (18.7)	63 (19.8)	2 (6.9)	
T3–4	282 (81.3)	255 (80.2)	27 (93.1)	

*BMI* body mass index, *NLR* neutrophil-to-lymphocyte ratio, *LNs* lymph nodes, *CEA* carcinoembryonic antigen, *CA19-9* carbohydrate antigen 199

### Regression analysis

Based on the results obtained from the comparison between groups, potentially important patient variables (*P* < 0.1) were included in the regression model, as shown in Table 2. Regression analysis indicated that CEA > 5 ng/ml (OR 2.894, 95% CI 1.158–7.230; *P* = 0.023), distance from anal verge > 9 cm (OR 2.406, 95% CI 1.019–5.681; *P* = 0.045) and poor differentiation (OR 9.712, 95% CI 3.302–28.651; *P* < 0.001) were significant risk factors for 253LN metastasis. It is worth noting that after regression analysis to control for confounding factors, distance from anal verge became an important predictor. However, preoperative serum CA19-9 and T stage did not seem to reliably predict 253LN metastasis.

**Table 2:** Multivariate regression analysis to determine independent predictive factors

Variable	Multivariate analysis	
	OR (95% CI)	*P* value
CEA (> 5 ng/ml vs. ≤ 5 ng/ml)	2.894 (1.158–7.230)	0.023
CA19-9 (> 33U/ml vs. ≤ 33 U/ml)	1.490 (0.581–3.824)	0.407
Distance from anal verge (> 9 cm vs. ≤ 9 cm)	2.406 (1.019–5.681)	0.045
Differentiation (poor vs. well/moderate)	9.712 (3.302–28.651)	< 0.001
T stage (T1–2 vs. ≤ T3–4)	2.204 (0.436–9.390)	0.368

*CEA* carcinoembryonic antigen, *CA19-9* carbohydrate antigen 199

### Establishment and verification of nomogram

**Fig. 3:**
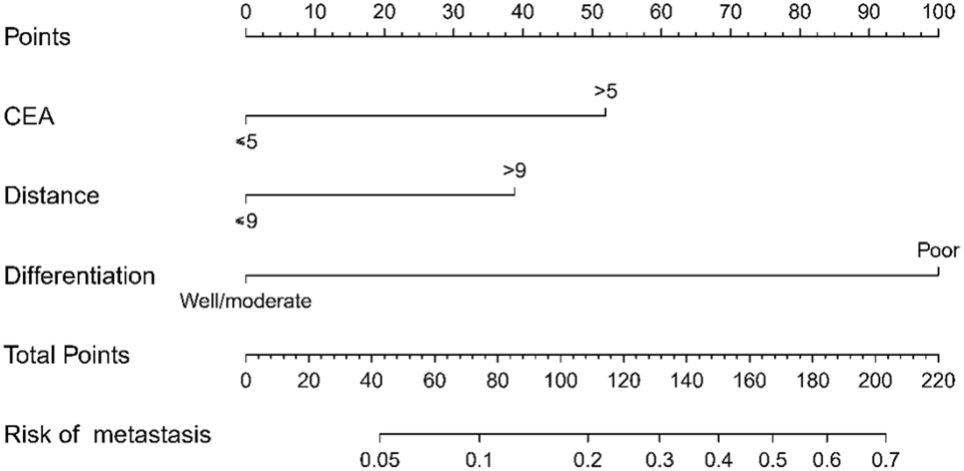
Nomogram for predicting the risk of 253LN metastasis in rectal cancer

Based on the three important predictors identified from regression analysis, a nomogram was developed, to determine the likelihood of 253LN metastasis in patients suffering from rectal cancer. The nomogram generated is displayed in Fig. 3. Each patient obtained a total score by adding the scores of the three predictors. For example, a case where preoperative serum CEA > 5 ng/ml, tumor distance from anal margin > 9 cm and differentiation was moderate attained a total score of 91 (52 + 39 + 0 = 91), indicating that the likelihood of 253LN metastasis was about 15%.

**Fig. 4:**
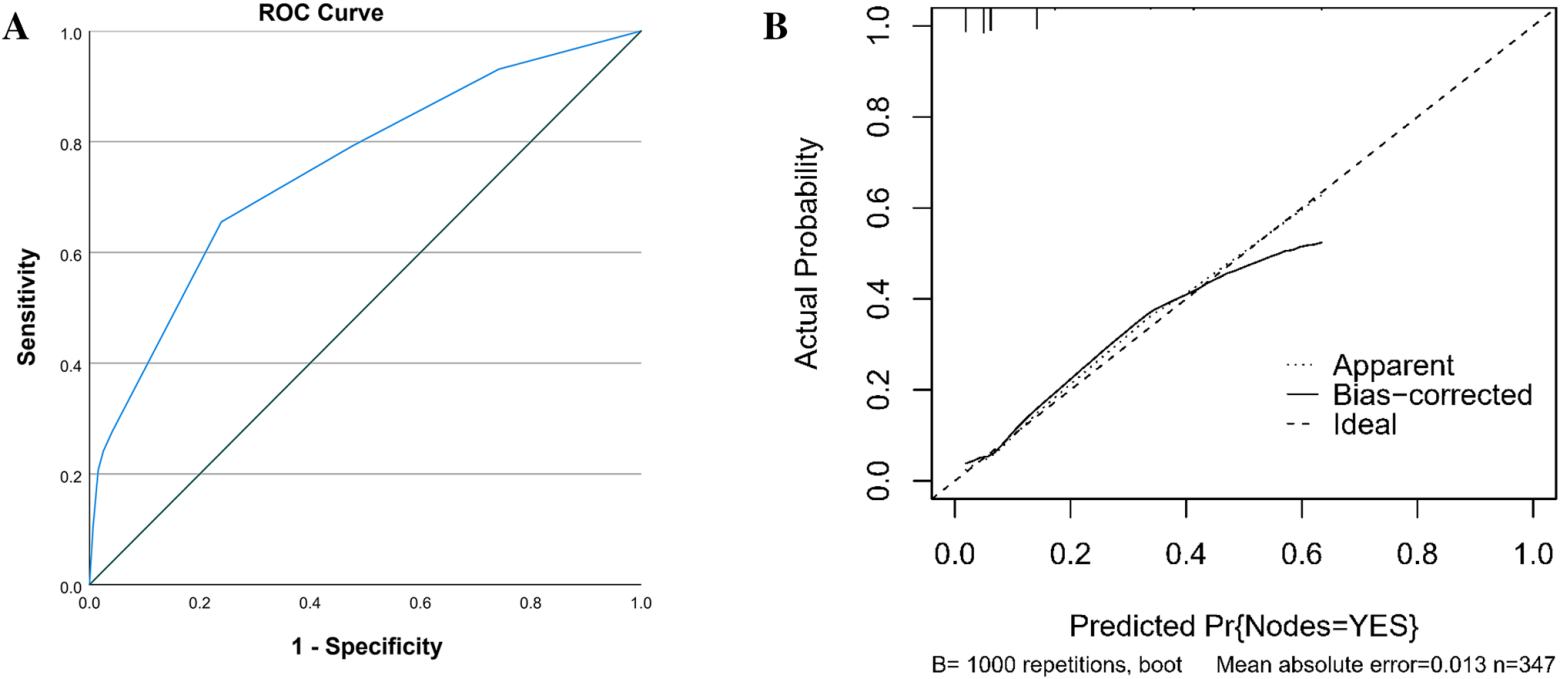
Validation of prediction model. **A** ROC curve of risk prediction model for 253LN metastasis of rectal cancer. AUC value was equal to 0.750 (95% CI 0.651–0.849). **B** Calibration curves of risk prediction model for 253LN metastasis of rectal cancer. The calibration showed moderate discrimination

The ROC curve generated for assessment of the nomogram’s predictive accuracy is illustrated in Fig. 4A. The area under curve (AUC) value of the nomogram was 0.750 (95% CI 0.651–0.849), thus validating its predictive power. According to the calibration of the nomogram curve (Fig. 4B) and Hosmer–Lemeshow test (*P* = 0.954), there was a good consistency between the prediction and observation probabilities, indicating that the nomogram was close to the ideal state.

**Fig. 5:**
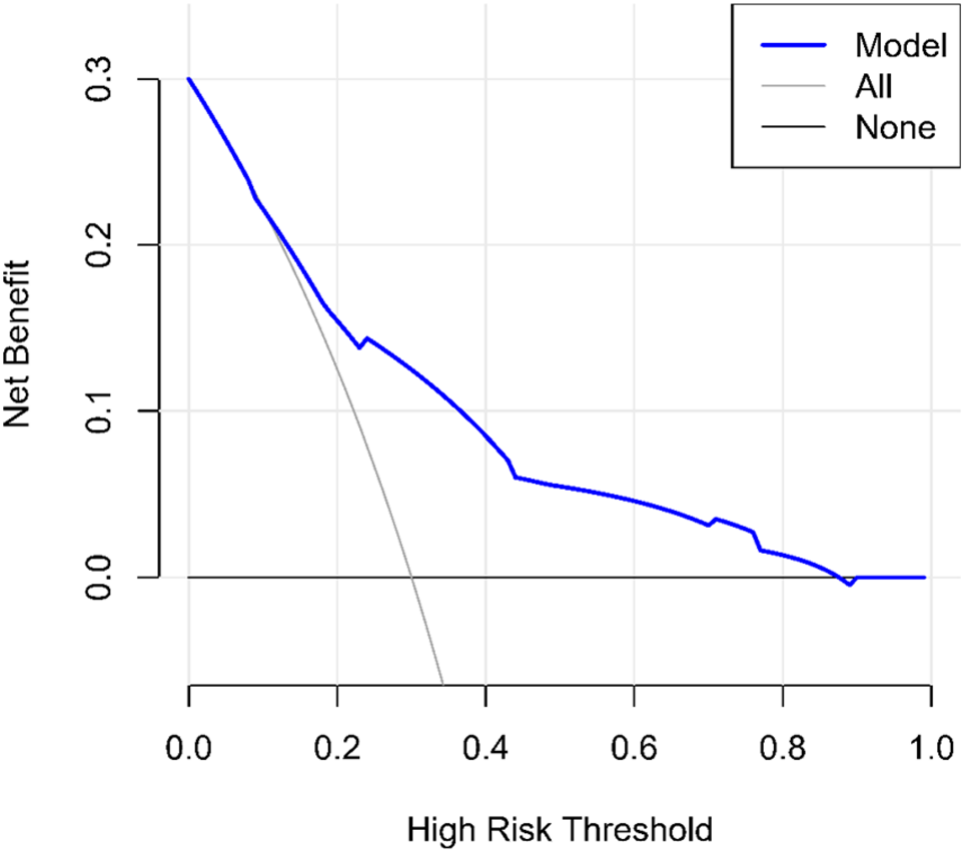
Decision curve analysis of 253LN metastasis risk prediction model for rectal cancer. The validity of the nomogram prediction model was verified

As shown in Fig. 5, DCA was used to evaluate the clinical utility of the nomogram developed. In situations where the threshold probability of the nomogram was between 12 and 87%, the nomogram provided a greater net benefit relative to making blanket decisions on surgical interventions. A threshold probability of 0.2 indicated that the nomogram provides a 15% net gain, proof of the appropriateness of the nomogram for practical settings.

## Discussion

A review of past and current literature revealed a shortfall of clinical investigations into 253LN metastasis. The development of a nomogram aimed at preoperative prediction of 253LN metastasis was the cornerstone of this study. The nomogram based on the data obtained before operation showed good precision and discrimination. In addition, DCA confirmed that the nomogram has good clinical utility. It was established that preoperative serum CEA, the distance between the tumor and the anal margin, and the degree of tumor differentiation were robust predictors of 253LN metastasis. Therefore, the nomogram developed can serve as reliable model for preoperative prediction of 253LN metastasis in rectal cancers.

Previous reports have described some potential risk factors for 253LN metastasis in colorectal cancer. In this study, preoperative serum CEA, degree of differentiation, and distance from the anal margin were identified as important predictors, a finding that was consistent with previous reports. Li et al. [3] considered that poor differentiation, preoperative T3–4 stage and ≥ 4 station 253 node retrieval were important risk factors for 253LN metastasis. A similar study on 253LN metastasis, performed by Chang et al. [11] focused on left colorectal cancer liver metastasis, but also investigated the prognostic value of 253LN. In contrast, this study focused specifically on preoperative prediction of 253 LN metastasis in rectal cancer.

According to the seventh edition of the American Joint Committee on cancer (AJCC) guidelines, para-aortic LN metastasis of rectal cancer was considered as systemic metastasis, whereas metastasis of the root LN of the IMA was defined as regional metastasis [9]. To date, the oncological and prognostic relevance of 253LN is still unclear, and the necessity of 253LN removal is still controversial [12]. Kang et al. [9] carried out a prospective study in 2011 involving 625 patients with stage III sigmoid colon or rectal cancer and concluded that 253LN metastasis was an important risk factor for systemic recurrence. Rao et al. [13] retrospectively analyzed 890 patients with left colon and rectal malignancies in 2018, and the results showed that 253 LN metastasis was an independent risk factor for poor prognosis. Therefore, although the incidence of 253LN metastasis reported in literature was relatively low, 253LN metastasis was still widely regarded as an important prognostic factor determining the survival of patients suffering from colorectal cancer [14]. However, thorough dissection of LN in the D3 region is particularly important for patients with existing 253LN metastasis.

In resection surgeries for colorectal cancer, the ligation level of the IMA can be divided into two categories: the starting point of the aorta, also known as HL, or the distal end of the starting point of the left colon artery, also known as LL [6]. The level of arterial ligation would affect urogenital function, LN output, perfusion of the distal artery, sympathetic nerve injury and anastomotic leakage [15]. The randomized controlled experiment conducted by Mari et al. [16] showed that LL of the IMA can better preserve the urogenital function without compromising the initial oncological results. Some meta-analyses suggest that HL increases the risk of anastomotic leakage [17]. However, most of the latest randomized controlled trials and meta-analyses suggest that LL does not impact the likelihood of surgical complications or cancer recurrence [16, 18–20].

The data used for the nomogram was routine and easily obtainable from a preoperative examination of the patient profile. It was observed that the metastasis rate of 253LN was significantly increased in patients where the distance between the tumor and the anal margin was > 9 cm. This was similar to the results of Jiang’s study that the metastasis rate of 253LN in sigmoid colon cancer was significantly higher than that in rectum, suggesting that the metastasis of 253LN may be related to the distance of the tumor [21]. Poor histological differentiation has always been considered as an important risk factor for LN metastasis [22]. In this study, poor differentiation is also strongly associated with increased 253LN metastases. Therefore, it can be safely stated that thorough removal of LN in the D3 area plays a critical role in the prognoses of patients whose cancers exhibit poor differentiation. Studies have shown that preoperative serum CEA can reliably predict metastatic disease and LN metastasis in patients with rectal cancer. Therefore, it is recommended that clinicians pay heed to the influence of preoperative serum CEA on tumor staging in rectal cancer [23, 24].

The regression model established had good predictive performance, but the nomogram was unable to portray the clinical consequences of false calibration or specific level discrimination. DCA is a tool that can be beneficial in such situations, identifying the optimal method on the basis of true clinical value [25]. According to DCA, this nomogram can obtain better benefits in a relatively wide range of probabilities, enabling surgeons to objectively assess the need for total mesenteric lymphadenectomy. Preoperative identification of high risk patients using the nomogram proved to be pivotal for the decision to resect the LN in the affected area. However, it is plausible that the surgical team can make more accurate decisions using the developed nomogram in jointly with preoperative magnetic resonance imaging evaluation and intraoperative conditions.

The advantage of this study was that it focused on preoperative prediction of 253LN metastasis in rectal cancer. Patient data that easily obtained before surgery was analyzed and nomogram developed on the basis of robust predictor risk factors. However, as a retrospective analysis, this study still has various limitations. To begin with, this is a single center study with unavoidable potential bias. Second, the study only performed internal validation and did not validate external cohorts. Furthermore, a prognostic analysis on the patients in this study was not performed. It is imperative that a large scale prospective clinical studies involving rectal cancer are conducted to acquire strong scientific evidence.

## Conclusion

A nomogram to preoperatively predict LN metastasis in rectal cancer was developed and validated. This nomogram can be used at various stages of rectal cancer and is an essential part of decision making processes in rectal cancer treatment. Nonetheless, further external validation of this nomogram is required to appraise its suitability for clinical practice.

## Data Availability

The datasets used and/or analyzed during the current study are available from the corresponding author on reasonable request.
